# Supplementary figures and images for: Natural infection of parvovirus in wild fishing cats (Prionailurus viverrinus) reveals extant viral localization in kidneys
Source: PLoS One. 2021 Mar 2;16(3):e0247266. doi: 10.1371/journal.pone.0247266 (PMC7924760; doi:10.1371/journal.pone.0247266)

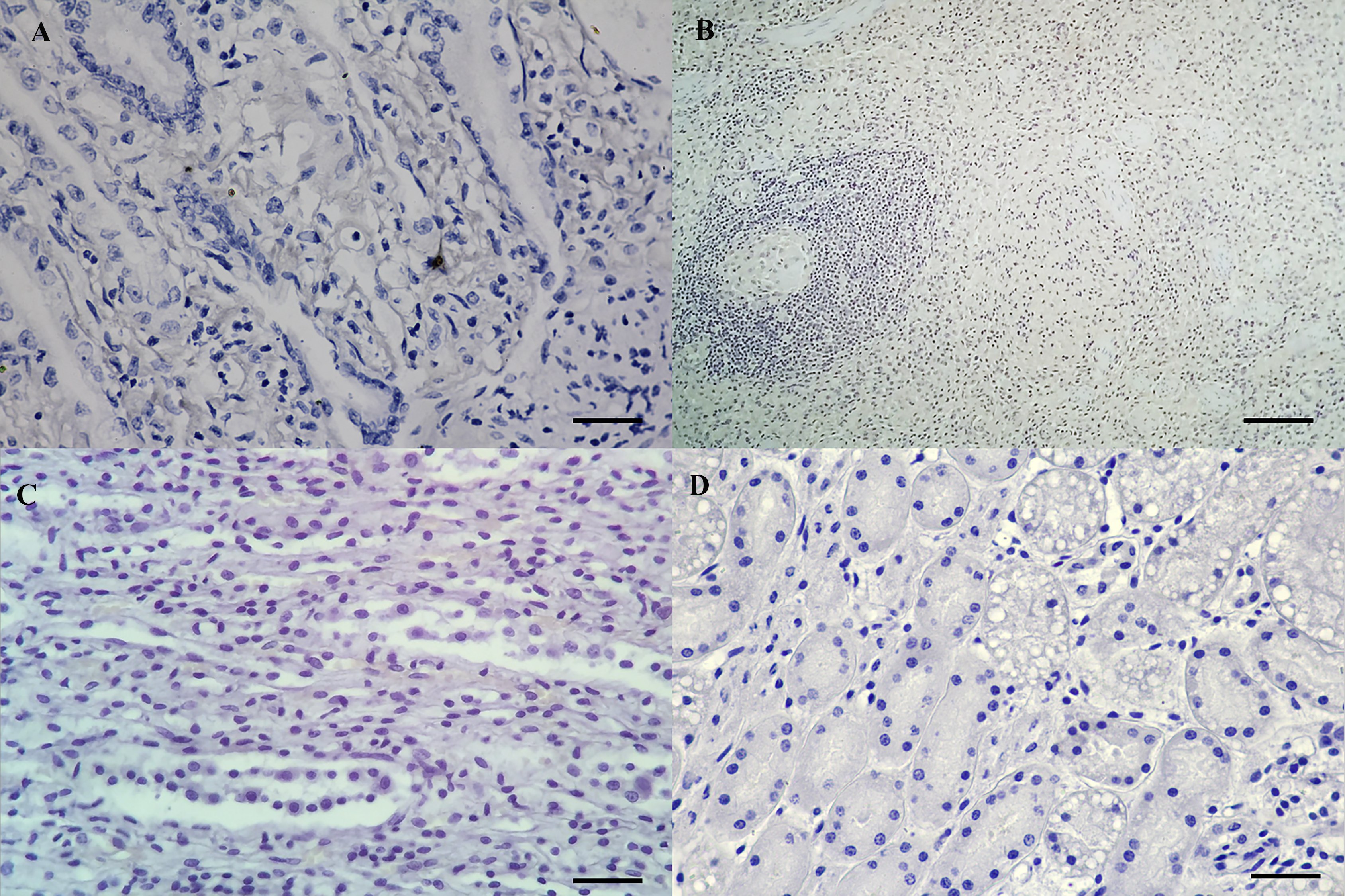

Supplement: S1 Fig — Photomicrograph of negative controls for CPPV-1 IHC (A-C) and CPPV-1 ISH (D). No CPPV-1 IHC reaction is present within the negative control section of (A) intestine, (B) spleen, and (C) kidney. (D) No immunoreactivity is present within a kidney section incubating with the TiLV probe (non-related probe). (TIF) [file pone.0247266.s001.tif]
