# Supplementary material for: Natural infection of parvovirus in wild fishing cats (Prionailurus viverrinus) reveals extant viral localization in kidneys
Source: PLoS One. 2021 Mar 2;16(3):e0247266. doi: 10.1371/journal.pone.0247266 (PMC7924760; doi:10.1371/journal.pone.0247266)
Supplement: S1 Table — (DOCX) [file pone.0247266.s002.docx]

**Natural infection of parvovirus in wild fishing cats (*Prionailurus viverrinus)* reveals extant viral localization in kidneys**

**S1 Table. List of wildlife carnivore species enrolled in the retrospective study of CPPV-1 detection.**

| **No.** | **Animal species** | **No.** | **Animal species** |
| --- | --- | --- | --- |
| 1. | *Aonyx cinereus* | 15. | *Panthera pardus* |
| 2. | *Prionailurus viverrinus* | 16. | *Panthera tigris* |
| 3. | *Paradoxurus hermaphroditus.* | 17. | *Panthera tigris tigris* |
| 4. | *Viverricula indica* | 18. | *Panthera leo* |
| 5. | *Herpestes javanicus* | 19. | *Neofelis nebulosa* |
| 6. | *Herpestes urva* | 20. | *Panthera pardus delacourii* |
| 7. | *Genetta genetta* | 21. | *Panthera leo nubica* |
| 8. | *Arctogalidia trivirgata* | 22. | *Arctocephalus pusillus* |
| 9. | *Paguma larvata* | 23. | *Prionailurus bengalensis* |
| 10. | *Otocyon megalotis* | 24. | *Prionailurus planiceps* |
| 11. | *Vulpes vulpes* | 25. | *Helarctos malayanus* |
| 12. | *Canis aureus* | 26. | *Crocuta crocuta)* |
| 13. | *Felis chaus* | 27. | *Vulpes zerda* |
| 14. | *Panthera tigris altaica* |  |  |
