# Supplementary material for: Natural infection of parvovirus in wild fishing cats (Prionailurus viverrinus) reveals extant viral localization in kidneys
Source: PLoS One. 2021 Mar 2;16(3):e0247266. doi: 10.1371/journal.pone.0247266 (PMC7924760; doi:10.1371/journal.pone.0247266)
Supplement: S1 File — (DOCX) [file pone.0247266.s003.docx]

**Natural infection of parvovirus in wild fishing cats (*Prionailurus viverrinus)* reveals extant viral localization in kidneys**

**Chutchai Piewbang^1,2^, Sabrina Wahyu Wardhani^2,3^, Jira Chanseanroj^4^, Jakarwan Yostawonkul^3,5^, Suwimon Boonrungsiman^5^, Nattika Saengkrit^5^, Piyaporn Kongmakee^6^, Wijit Banlunara^1^, Yong Poovorawan^4^, Tanit Kasantikul^7^, Somporn Techangamsuwan^1,2,*^**

**S1 File. Amplification procedures used for routine pan-virologic-family detection and full-length genetic characterization of fishing cat CPPV-1**

The extracted nucleic acids derived from each sample of three fishing cats were individually subjected for RT-PCR and conventional PCR amplifications using a Qiagen OneStep RT-PCR kit (Qiagen GmbH, Hilden, Germany) and a GoTaq^®^ Hot Start Green Master Mix (Promega, Madison, WI, U.S.A.), respectively as a master mix kit. The RT-PCR was performed to detect the paramyxovirus, pneumovirus, calicivirus, influenza virus, and coronavirus, while the conventional PCR was used for detection of herpesvirus, bocavirus and parvovirus. PCR reagents and cycling condition of each PCR was retrieved from previous publications and summarized in Table 1. The non-template samples were served as negative controls.

**Table 1**. The amplification procedures and positive controls used in the pan-virologic-family PCR for routine virus screening.

| **Virologic-family detection** | **Amplifications procedure** | | | **Positive control** | **Reference for positive controls** |
| --- | --- | --- | --- | --- | --- |
|  | **Reaction** | **Cycling no.** | **Annealing (^◦^C)** |  |  |
| Herpesvirus | PCR | 45 | 46 | CaHV-1 | (1) |
| Bocavirus | PCR | 40 | 50 | FBoV-1 | (2) |
| Parvovirus | PCR | 40 | 55 | CPV-2c | (3) |
| Paramyxovirus | RT-PCR | 40 | 41 | CDV | (4) |
| Pneumovirus | RT-PCR | 40 | 49.6 | CnPnV | (5) |
| Calicivirus | RT-PCR | 40 | 49 | FCV | (2) |
| Influenza virus | RT-PCR | 40 | 48 | CIV H3N2 | (6) |
| Coronavirus | RT-PCR | 40 | 54 | CRCoV | (6) |

To characterize the coding sequence of the fishing cat CPPV-1, the primers were retrieved from a previous publication (2) and described in Table 2. Cycling conditions were performed according to the pan-parvovirus PCR protocols as described in Table 1.

**Table 2.** The CPPV-1 Specific primers use for genetic sequencing of coding sequences of fishing cat CPPV-1 in this study. The primers were derived from previous publication (2)

| Primer name | Sequence (5’-3’) |
| --- | --- |
| FPV-NS-Fext | GACCGTTACTGACATTCGCTTC |
| FPV-NS-Rext | GAAGGGTTAGTTGGTTCTCC |
| FPV-NS-Fint | GTTGAAACCACAGTGACGACAG |
| FPV-NS-Rint | CATCATCCARTCTTCAGGTG |
| FPV-2161F | TTGGCGTTACTCACAAAGACGTRC |
| FPV-3475R | GTTGGTGTGCCACTAGTTCCAGTA |

1. Piewbang C, Rungsipipat A, Poovorawan Y, Techangamsuwan S. Viral molecular and pathological investigations of Canid herpesvirus 1 infection associated respiratory disease and acute death in dogs. Acta Vet Brno. 2017;67(1):11.

2. Piewbang C, Kasantikul T, Pringproa K, Techangamsuwan S. Feline bocavirus-1 associated with outbreaks of hemorrhagic enteritis in household cats: potential first evidence of a pathological role, viral tropism and natural genetic recombination. Sci Rep. 2019;9(1):16367.

3. Nguyen Manh T, Piewbang C, Rungsipipat A, Techangamsuwan S. Molecular and phylogenetic analysis of Vietnamese canine parvovirus 2C originated from dogs reveals a new Asia-IV clade. Transbound Emerg Dis. 2020.

4. Piewbang C, Radtanakatikanon A, Puenpa J, Poovorawan Y, Techangamsuwan S. Genetic and evolutionary analysis of a new Asia-4 lineage and naturally recombinant canine distemper virus strains from Thailand. Sci Rep. 2019;9(1):3198.

5. Piewbang C, Techangamsuwan S. Phylogenetic evidence of a novel lineage of canine pneumovirus and a naturally recombinant strain isolated from dogs with respiratory illness in Thailand. BMC Vet Res. 2019;15(1):300.

6. Piewbang C, Rungsipipat A, Poovorawan Y, Techangamsuwan S. Cross-sectional investigation and risk factor analysis of community-acquired and hospital-associated canine viral infectious respiratory disease complex. Heliyon. 2019;5(11):e02726.
