# Supplementary material for: Natural infection of parvovirus in wild fishing cats (Prionailurus viverrinus) reveals extant viral localization in kidneys
Source: PLoS One. 2021 Mar 2;16(3):e0247266. doi: 10.1371/journal.pone.0247266 (PMC7924760; doi:10.1371/journal.pone.0247266)
Supplement: Attachment — Submitted filename: Rebuttal response PONE-D-20-33818.docx [file pone.0247266.s005.docx]

**Rebuttal response on PONE-D-20-33818**

**Journal requirements:**

When submitting your revision, we need you to address these additional requirements.

*1. Please ensure that your manuscript meets PLOS ONE's style requirements, including those for file naming. The PLOS ONE style templates can be found at*

*https://journals.plos.org/plosone/s/file?id=wjVg/PLOSOne_formatting_sample_main_body.pdf and*

[*https://journals.plos.org/plosone/s/file?id=ba62/PLOSOne_formatting_sample_title_authors_affiliations.pdf*](https://journals.plos.org/plosone/s/file?id=ba62/PLOSOne_formatting_sample_title_authors_affiliations.pdf)

**Response**: We have revised the manuscript following PLOS ONE's style requirements.

*2. To comply with PLOS ONE submissions requirements, please provide methods of sacrifice in the Methods section of your manuscript.*

**Response**: The three fishing cats that enrolled in this study were naturally died by first 2 fishing cats were found dead and another fishing cats died during referral. Thus, we have no information regarding the method of sacrifice. Details of animals in this study were described in Materials and Methods section.

**Reviewers' comments:**

**Reviewer's Responses to Questions**

**Comments to the Author**

1. Is the manuscript technically sound, and do the data support the conclusions?

The manuscript must describe a technically sound piece of scientific research with data that supports the conclusions. Experiments must have been conducted rigorously, with appropriate controls, replication, and sample sizes. The conclusions must be drawn appropriately based on the data presented.

Reviewer #1: Partly

Reviewer #2: Yes

2. Has the statistical analysis been performed appropriately and rigorously?

Reviewer #1: I Don't Know

Reviewer #2: N/A

3. Have the authors made all data underlying the findings in their manuscript fully available?

The [PLOS Data policy](https://eur05.safelinks.protection.outlook.com/?url=http%3A%2F%2Fwww.plosone.org%2Fstatic%2Fpolicies.action%23sharing&data=04%7C01%7C%7Ca867ce0170bb4449bb4e08d88fd26d6f%7C84df9e7fe9f640afb435aaaaaaaaaaaa%7C1%7C0%7C637417481223596792%7CUnknown%7CTWFpbGZsb3d8eyJWIjoiMC4wLjAwMDAiLCJQIjoiV2luMzIiLCJBTiI6Ik1haWwiLCJXVCI6Mn0%3D%7C1000&sdata=mVN6WPfxh2VIgGWgCPcXIybwqhml0E8sGKwh8Gyjgl4%3D&reserved=0) requires authors to make all data underlying the findings described in their manuscript fully available without restriction, with rare exception (please refer to the Data Availability Statement in the manuscript PDF file). The data should be provided as part of the manuscript or its supporting information, or deposited to a public repository. For example, in addition to summary statistics, the data points behind means, medians and variance measures should be available. If there are restrictions on publicly sharing data—e.g. participant privacy or use of data from a third party—those must be specified.

Reviewer #1: Yes

Reviewer #2: Yes

4. Is the manuscript presented in an intelligible fashion and written in standard English?

PLOS ONE does not copyedit accepted manuscripts, so the language in submitted articles must be clear, correct, and unambiguous. Any typographical or grammatical errors should be corrected at revision, so please note any specific errors here.

Reviewer #1: Yes

Reviewer #2: Yes

5. Review Comments to the Author

Please use the space provided to explain your answers to the questions above. You may also include additional comments for the author, including concerns about dual publication, research ethics, or publication ethics. (Please upload your review as an attachment if it exceeds 20,000 characters)

***Reviewer #1:*** *The authors investigated three cases of natural canine carnivore protoparvovirus-1 (CPPV-1) infection in wild fishing cats. In their article they describe a new tropism of parvovirus to renal tissue by using PCR, immunohistochemistry and transmission electron microscopy.*

Major comments:
*1. In the materials and methods (Line 116ff) the authors describe that they isolate nucleic acids from fresh samples. Since the authors also claim a high degree of autolysis in cases 1&2 they have to specify: 1.) nucleic acids were isolated from which organs in which cats, and 2.) PCRs for virus families were performed on which organs from which cats.*

**Response:** With the request from reviewer, we have provided additional information of the sample collection by describing details as **“The fresh tissue samples including heart, lung, liver, spleen, and kidney of all fishing cats plus additional intestinal and mesenteric lymph node tissues of fishing cat no. 3, were subjected to viral nucleic acid extraction**.” Page 6, lines 113-115. For additional information of PCRs for virus families, we have added more details regarding the PCR protocols in S1_File.

*2. The authors describe that they perform pan-virologic-family PCRs - they should specify for which families they used PCR and for which RT-PCR.*

**Response:** We have added more details in S1_file.

*3. In addition, the used PCR/RT-PCR kits have to be specified including PCR conditions and applied positive controls for PCR/RT-PCR.*

**Response:** We have provided more details as description in S1_file.

*4. Where process controls included during nucleic acid extraction since autolysis may have a significant impact on the performance of the PCR reaction.*

**Response:** Thank you for your concern. At this point, we do respect that the autolysis tissues may significantly impact on the performance of the PCRs, thus the tissue samples which revealed moderate to marked autolysis (presented on necropsy desk) would be not included for the molecular study. Furthermore, we have validated all extracted nucleic acids by further performing the PCR to detect the glyceraldehyde-3-phosphate dehydrogenase (GAPDH) gene as an internal housekeeping gene in order to validate the performance of samples and extraction process. Thus, we have provided the information as “**The PCR protocols, reagents, cycling conditions and positive/ negative controls used in the reactions are described in the S1 File. PCR detection specific of the glyceraldehyde-3-phosphate dehydrogenase (GAPDH) gene was used as an internal control as described previously (31).**” In page 6, lines 124-127.

*5. Regarding the postmortem findings (lines 214ff) the authors stated that "Other organs showed advanced autolysis and so they could not be investigated." Do the authors mean by histology, immunohistochemistry, PCR, TEM? Please specify. In the following sentence the authors state that histologically all fishing cats showed desquamative enteritis, lymphoid depletion in lymph nodes and spleen. What does this mean in the context of the sentence before? Please include a picture detailing lymphoid depletion in spleen and lymph nodes. Were these findings associated with a positive signal in PCR and/or IHC for CPPV-1?*

**Response:** Thank you for question regarding the postmortem findings that the presented statements may lead confusion. Due to the fact that fishing cats no. 1&2 have been found dead and the most of organs revealed autolysis, which is presenting on necropsy desk, resulting in other organs could not be investigated. Thus, we do *delete* “Other organs showed advanced autolysis and so they could not be investigated” and revise the statement regarding the post-mortem findings as “**All necropsied fishing cats were moderately emaciated with varying degrees of dehydration while 2 of the 3 fishing cats (case nos. 1 and 2) showed moderate autolysis. Prominently, the spleen and kidneys were congested in all fishing cats. Note that fishing cat no.3 had macroscopic lesions of catarrhal enteritis that contained a watery brown-yellowish content in their lumens and congestion of the mesenteric lymph node.**” in page 11, lines 236-240. Furthermore, we have provided the H&E and CPPV-1 IHC pictures of lymph node as your requested in **Fig 1 and the IHC-positive signals were identified in the area of lymphoid necrosis as described in Fig 1 legend.**

*6. Line 230f: What do you mean with severely collapsed intestinal mucosa?*

**Response:** We have revised the sentence as **“(B) Fishing cat no. 3. The intestinal mucosa is diffusely and severely desquamated, the intestinal villi are mostly short, many crypts are lost or contain karyorrhectic debris (inset) and there are decreased numbers of goblet cells**.”. Fig1 legend. (Page 12, lines 254-256.)

7. Regarding the tubular necrosis: Do the authors perform PAS-stain to exclude hypoxia as being the cause of the tubular necrosis? Were the necrotic cells positive for CPPV-1?

**Response:** We have performed the PAS staining in kidney section of all fishing cats. The results were shown in Fig 2B. and described as **“Regarding the severe renal tubular necrosis, we further performed PAS staining in kidney sections to demonstrate the cellular architecture of kidney tissue and to exclude systemic hypoxia as being the possible cause of this lesion. PAS staining demonstrated the disruption of renal tubular epitheliums while the architectures of most renal tubular basement membranes were intact (Fig 2B).**” in Result section (pages 12-13, lines 264-268) and “**we performed PAS staining to demonstrate the cellular architecture of renal tissue and to exclude systemic hypoxia as being the possible cause of tubular necrosis. PAS staining revealed disruption of tubular epithelium while most tubular basement membranes were intact, suggesting that this lesion may not result from systemic hypoxia (51)**.” in Discussion section (Page 21, lines 406-409). Of note. The CPPV-1 IHC signals were frequently seen in the area of tubular necrosis as we have revised as **“The CPPV-1 ISH-immunoreactivity was positive in all fishing cats and diffusely localized in the nucleus of renal tubular epithelial cells, where the tubular necrosis lesions were observed (Fig 2D).”** in Results section (page 15, lines 275-277).
8. The IHC-picture of the kidney looks like as if almost all renal tubular cells were positive for CPPV-1 antigen. To confirm their finding the authors should perform in situ hybridization to show a correlation/specificity of the IHC for CPPV-1.

**Response:** We performed the ISH in kidney sections, derived from all fishing cats and the result picture was shown in Fig 2D. Details regarding the protocols and results were described in Materials and Methods section (pages 7-8, lines 153-171) and Results section (page 13, lines 275-278, respectively.

*9. Regarding the retrospective study (lines 315ff): What do the authors mean by "A retrospective study of 136 zoo-wild animals in 27 carnivores revealed the presence of CPPV-1 genomic antigen."? Does this mean 27 cases were positive? The authors should explain which organs were investigated in which animal and what methods including nucleic acid extraction and PCR method were used. Do the authors also perform IHC? If all samples were negative, how can the authors show that this result is not related to tissue storage / fixation?*

**Response:** The statement regarding the result of retrospective study is confused so we revised the sentence as “**A retrospective study of 136 zoo-wild animals derived from 27 different carnivores did not reveal the presence of CPPV-1 genomic antigen as detected by PCR**” in page 19, lines 360-361. For the methods and samples used for retrospective study, we have provided the information in the Materials and Methods section as “305 selective fresh samples were included for genomic extraction and identification targeting of the CPPV-1 capsid gene as described above.” in page 11, lines 229-231. Furthermore, we have revised the discussion as the prolonged sample storage may affect the PCR result in as **“The negative evidence of retrospective CPPV-1 detection in zoo-wild samples in this study may result from either no close-contact among susceptible animals or, on the other hand, prolonged sample storage may affect the stability of genomic materials.**.**”** in page 22, lines 432-434.

Minor comments:
*Line 90 "... CPPV-1 reveals FPV is a ...": please change is to as
Line 327 "... and tropism and in kidney...": please delete the second "and".*

**Response:** We have revised them as reviewer suggestions.

Reviewer #2: Carnivore protoparvovirus-1 (CPPV-1), include feline panleukopenia virus (FPV) and canine parvovirus (CPV), which are widespred among domestic and wild carnivores, causing systemic fatal diseases. Wild fishing cats (Prionailurus viverrinus), is a vulnerable species. Virological (PCR and TEM) and gross and microscopic investigations, identified the presence of an FPV-like parvovirus in fishing cats found dead. Postmortem examination revealed severe enteritis, lymphadenopathy and nephritis. On whole genome sequencing, the virus closely resembled FPV sequences with two peculiar amino acid mutations I566M and M569R in the capsid protein.

*The manuscript is of relevant scientific interest. The introduction is well written. The materials and methods seem adequate. The discussion is correct and rather balanced. I have only minor comments.*

**Response:** Thank you for your feeling positive with our manuscripts.

i) there is a confusion with the terms CPPV-1 and FPV, that are nearly the same thing. I would suggest to use consistently the term FPV-like parvovirus

**Response:** We have revised as reviewer suggestion.

ii) there are some parts of the manuscript that could be deleted, reworded or re-phrased.
Abstract: check English. Shorten the final part, very generic.
Line 81-83 it is not clear
Lines 81-88: rephrase
Line 90
Line 316
Line 322: please delete/replace the semicoma (;) after infection
Line 325: FPV is not a variant of CPPV-1
Line 346-347: I would delete the comments (Suggesting… Therefore….)
Line 360-361: rephrase
Line 363: odd sentence (Supporting viral genomic detection…). Rephrase

**Response:** We have revised as reviewer suggestions.

iii) A table with the list (and details) of primers used (consensus and specific), even as supplemental file, should be added.

**Response:** Details of primers used in this study were described in S1_File.

iv) General comment: the English is good but should be further refined.

**Response:** The English have been reviewed and revised by native speakers.

6. PLOS authors have the option to publish the peer review history of their article ([what does this mean?](https://eur05.safelinks.protection.outlook.com/?url=https%3A%2F%2Fjournals.plos.org%2Fplosone%2Fs%2Feditorial-and-peer-review-process%23loc-peer-review-history&data=04%7C01%7C%7Ca867ce0170bb4449bb4e08d88fd26d6f%7C84df9e7fe9f640afb435aaaaaaaaaaaa%7C1%7C0%7C637417481223606787%7CUnknown%7CTWFpbGZsb3d8eyJWIjoiMC4wLjAwMDAiLCJQIjoiV2luMzIiLCJBTiI6Ik1haWwiLCJXVCI6Mn0%3D%7C1000&sdata=FUp0EuQ5ZN%2F0xAfNmkWSQHFcU7iR9N00ZH42izJb3z4%3D&reserved=0)). If published, this will include your full peer review and any attached files.


If you choose “no”, your identity will remain anonymous but your review may still be made public.

**Response:** On behalf of corresponding author, I do agree to make public revision.


**Do you want your identity to be public for this peer review?** For information about this choice, including consent withdrawal, please see our [Privacy Policy](https://eur05.safelinks.protection.outlook.com/?url=https%3A%2F%2Fwww.plos.org%2Fprivacy-policy&data=04%7C01%7C%7Ca867ce0170bb4449bb4e08d88fd26d6f%7C84df9e7fe9f640afb435aaaaaaaaaaaa%7C1%7C0%7C637417481223606787%7CUnknown%7CTWFpbGZsb3d8eyJWIjoiMC4wLjAwMDAiLCJQIjoiV2luMzIiLCJBTiI6Ik1haWwiLCJXVCI6Mn0%3D%7C1000&sdata=Ka1STlmAEj%2FWvKY2oTdzR0meutBVDgoxL8QlmzuxRxU%3D&reserved=0).

Reviewer #1: No

Reviewer #2: No
