# Supplementary material for: Natural infection of parvovirus in wild fishing cats (Prionailurus viverrinus) reveals extant viral localization in kidneys
Source: PLoS One. 2021 Mar 2;16(3):e0247266. doi: 10.1371/journal.pone.0247266 (PMC7924760; doi:10.1371/journal.pone.0247266)
Supplement: Attachment — Submitted filename: Rebuttal response on PONE-D-20-3318R2.docx [file pone.0247266.s006.docx]

**Rebuttal response on PONE-D-20-33818R2**

**Reviewer #1**: *The authors did a great job and significantly improved the manuscript. However, there are still some issues:

In the materials and methods (Animals and routine postmortem examination section) the authors state: "Selective vital organs including lung, liver, heart, spleen, and kidney were sampled from all fishing cats, while the intestine and mesenteric lymph node were additionally collected from fishing cat no.3." while in the (full-length genetic characterization of fishing cat CPPV-1) section the authors write: "Briefly, the extracted nucleic acids obtained from intestine, spleen and kidneys of two fishing cats were individually amplified using a GoTaq® Hot Start Green Master Mix (Promega, Madison, WI, U.S.A.) and specific primers (S1 File)."
--> How can you isolate nucleic acids from the intestine of two cats when samples are only available from cat 3? Please clarify.*

**Response:** Thank you for your positive review on our manuscript. There is a missing information regarding the samples used for genome sequencing and we do revise them as “the extracted nucleic acids obtained from spleen and kidneys of two fishing cats plus additional intestinal tissue of fishing cat no. 3, were…” in page 9, lines 183-184.

*In the description of figure 1 the size of the scale bars is missing.*

**Response:** the size of the scale bars in Fig. 1 is already described as found in page 13, line 276.  *In the abstract in line 29 I suggest to change ... revealed lesions of intestine ... to revealed lesions in intestine ...*

**Response: We revised it as reviewer suggestion.**

**Reviewer #3:** *The manuscript is much improved from a previous version, and the pathology images are much better. A few minor issues are detailed below, but I fully expect that this can be accepted when these minor issues are addressed

Line 26- are widely spread (add in word)
Line 27- a globally vulnerable …. (reword)
Line 30- infection by the virus (reword)*

**Response: Thank you for your recommendation, we have revised them as reviewer suggestions.**

*Line 67- this needs a reference, and some good references here for cats infected with CPV- both symptomatic and asymptomatic*

**Response:** We have provided the potential references as your suggestion as found in page 3, line 67.

*Line 81- replace of, with as the
Within your materials and methods you take some samples from some animals, but not from others, yet the samples not taken appear later in the study for the PCR analysis. Can you please check this just for clarity?*

**Response:** There is some missing information regarding the samples used for genome sequencing of the CPPV-1, so we have revised the sentence as “the extracted nucleic acids obtained from spleen and kidneys of two fishing cats plus additional intestinal tissue of fishing cat no. 3, were…” in page 9, lines 183-184.

*Line 408- determinant may sound better than determination?*

**Response:** We revised it as reviewer suggestion.
